# Delayed-Onset Localized Bullous Pemphigoid on an Umbilical Hernia Repair Scar: An Example of Ruocco's Immunocompromised Cutaneous District

**DOI:** 10.7759/cureus.113453

**Published:** 2026-07-27

**Authors:** Ergün Tercan, Tubanur Cetinarslan, Peyker Temiz, Sule Yildiz Sagcan Tercan, Aylin Türel Ermertcan

**Affiliations:** 1 Pathology, Manisa Merkez Efendi State Hospital, Manisa, TUR; 2 Dermatology, Manisa Celal Bayar University Faculty of Medicine, Manisa, TUR; 3 Pathology, Manisa Celal Bayar University Faculty of Medicine, Manisa, TUR

**Keywords:** autoimmune blistering disorder, localized bullous pemphigoid, ruocco’s immunocompromised cutaneous district, surgical scar, umbilical hernia repair

## Abstract

Bullous pemphigoid (BP) represents the most prevalent autoimmune subepidermal blistering condition, disproportionately affecting the elderly and frequently manifesting as a widespread disease. Localized variants are considerably rare and tend to emerge at sites subjected to prior trauma, surgical intervention, or persistent inflammation. Herein, we describe a 62-year-old male who developed bullous lesions restricted to an umbilical hernia repair scar, with onset occurring 15 years following open surgical repair involving prosthetic mesh. Clinical evaluation revealed a single, intact linear bulla along the scar without any mucosal or systemic involvement; the Nikolsky sign was absent. Histopathological analysis identified a subepidermal blistering pattern, and direct immunofluorescence demonstrated linear IgG deposits along the dermoepidermal junction, confirming the diagnosis of BP. Treatment with topical corticosteroids resulted in full resolution within approximately two weeks, with no recurrence detected during the follow-up period. Review of previously reported surgically triggered localized BP cases indicates that a 15-year latency is exceeded by only two prior reports, both at non-abdominal sites, underscoring the exceptional duration observed here. The extended latency period observed in this case is consistent with the concept of an immunocompromised cutaneous district, wherein localized immune dysregulation may persist well beyond the initial tissue insult. To the best of our knowledge, this constitutes the first reported case of localized BP arising on an umbilical hernia repair scar.

## Introduction

Bullous pemphigoid (BP) is the most frequently encountered autoimmune subepidermal blistering disorder, with a predilection for older individuals. It is characterized by autoantibodies directed against the hemidesmosomal proteins BP180 and BP230, leading to separation at the dermoepidermal junction and the formation of tense subepidermal blisters [1]. The disease typically follows a chronic, relapsing course punctuated by alternating periods of flare and remission [2]. While the generalized form remains the classic presentation, localized variants have been increasingly recognized in the literature and are estimated to account for roughly 16-29% of all BP cases [3]. The differential diagnosis of localized BP should encompass a spectrum of vesiculobullous and inflammatory dermatoses, including epidermolysis bullosa acquisita, bullous impetigo, contact dermatitis, bullous lichen planus, pemphigus vulgaris, erythema multiforme with bullous features, bullous fixed drug eruption, and infectious etiologies such as herpes simplex virus and varicella-zoster virus [4]. Localized BP following surgical procedures has been documented in the setting of various operations, including incisional herniorrhaphy, open reduction and internal fixation of skeletal fractures, amputation, and cholecystectomy [5,6]. These cases have been explained by the concept of the immunocompromised cutaneous district, whereby previously injured skin becomes more susceptible to the development of immune-mediated disorders [7]. Recent evidence further supports surgery and cutaneous trauma as important triggers of localized bullous pemphigoid [8]. However, delayed-onset localized BP developing many years after surgery is uncommon and may present a diagnostic challenge because of its restricted distribution and prolonged latency. In the present report, we describe a patient who developed localized BP over an umbilical hernia repair scar more than 15 years after surgery, representing an unusual example of an immunocompromised cutaneous district.

## Case presentation

**Figure 1:**
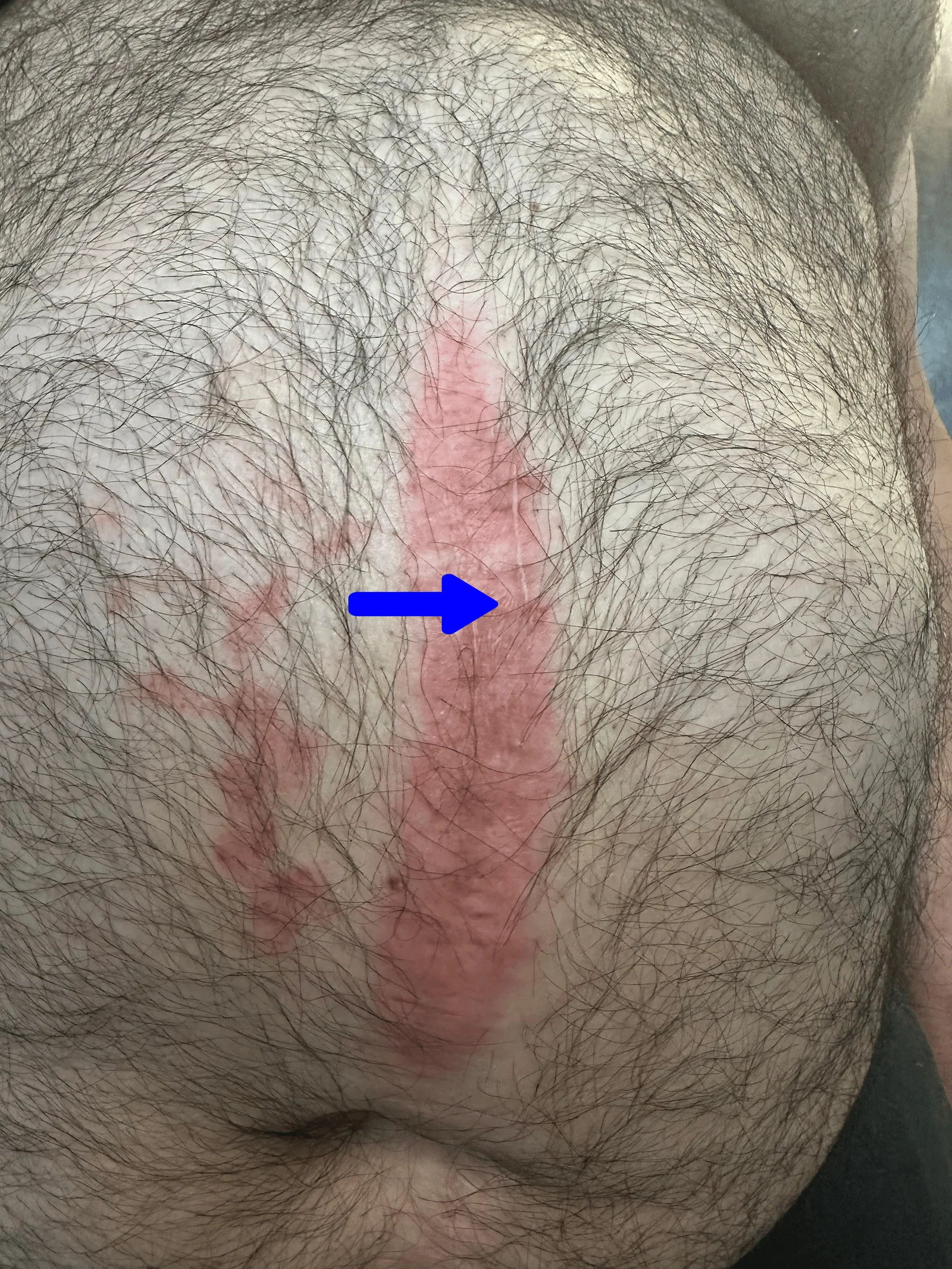
An intact bulla measuring 3 × 2 cm is located on a 12-cm linear umbilical hernia repair scar (blue arrow).

A 62-year-old male patient was evaluated for a three-day history of pruritic blistering lesions confined to a prior surgical scar. Fifteen years before this presentation, he had undergone open repair of an umbilical hernia utilizing prosthetic mesh. The patient denied any prior dermatological symptoms or recent medication changes. The patient had a history of hypertension, for which he had been receiving long-term perindopril therapy. No other significant medical history was reported. Dermatological examination demonstrated a solitary, flaccid, intact, 3 × 2 cm bulla arranged linearly along the scar margin (Figure 1).

**Figure 2:**
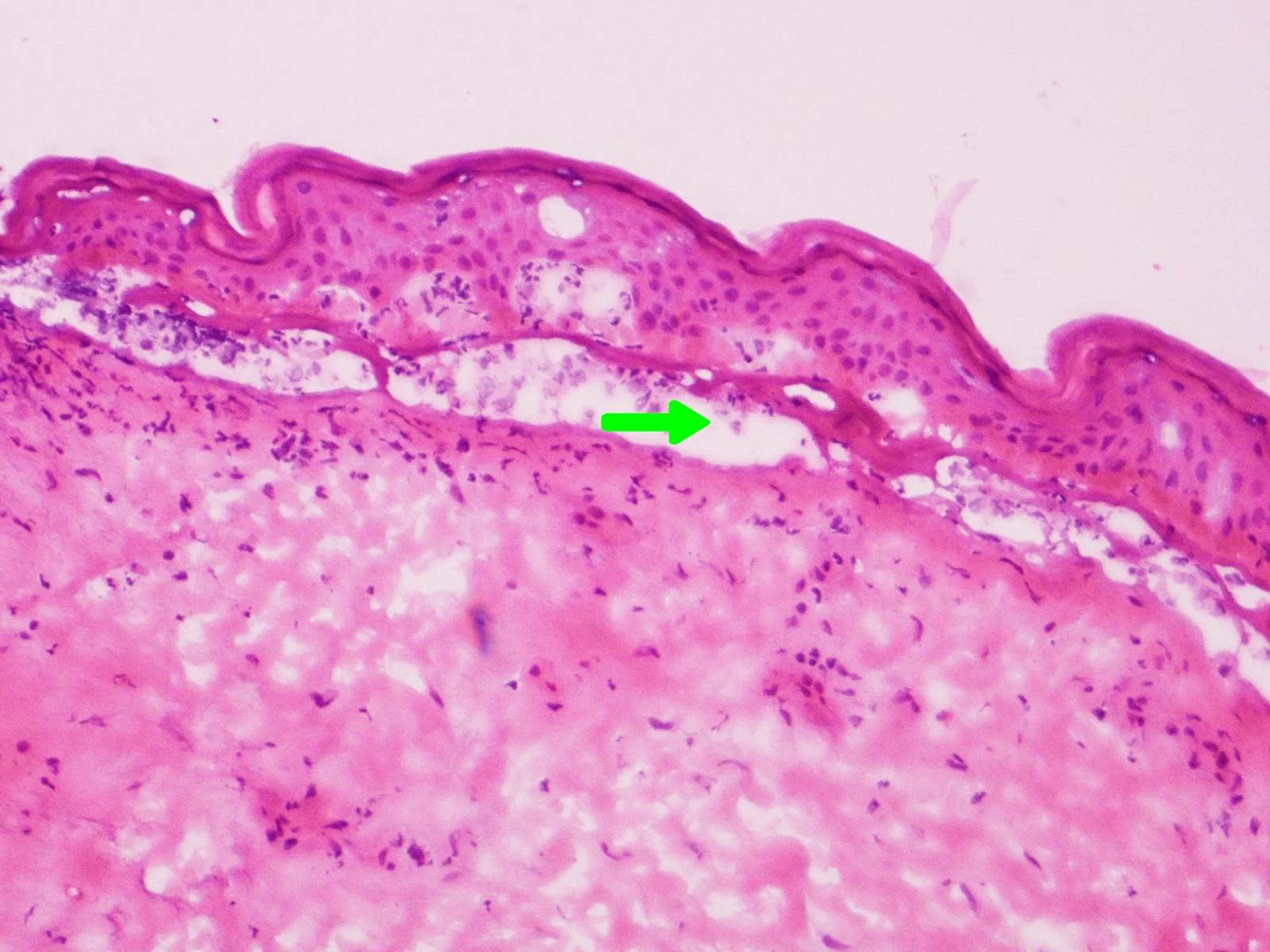
Subepidermal separation and bullous formation (green arrow) (hematoxylin and eosin staining x40).

**Figure 3:**
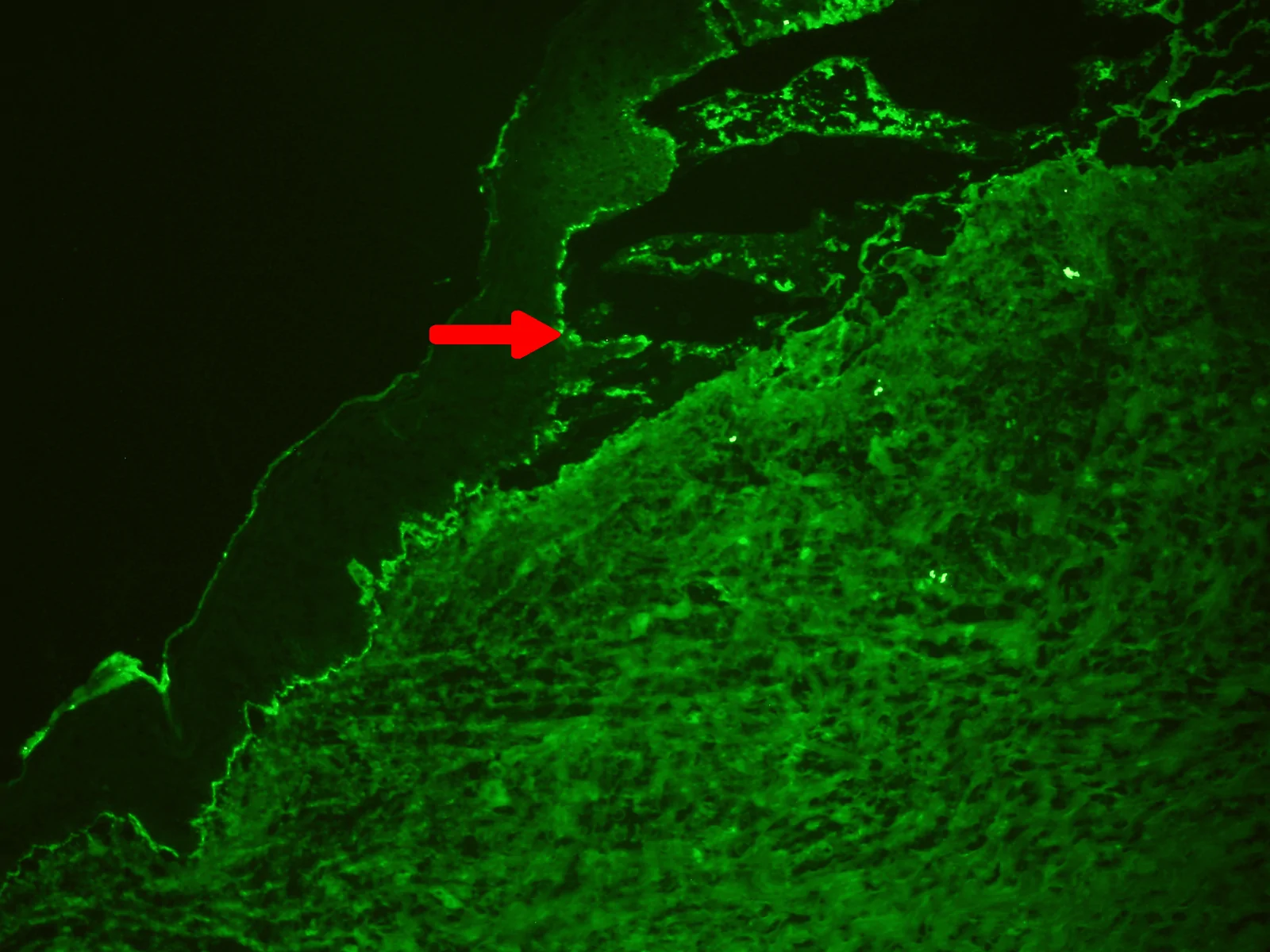
Direct immunofluorescence shows linear IgG deposition at the dermoepidermal junction (red arrow) (x100).

The Nikolsky sign was not elicited. No bullae or erosions were identified at other cutaneous or mucosal sites. Initial differential considerations included epidermolysis bullosa acquisita, bullous allergic contact dermatitis, bullous impetigo, and bullous pemphigoid. Histopathological assessment of a punch biopsy specimen confirmed subepidermal separation with bullous formation. Direct immunofluorescence studies revealed linear IgG deposition along the dermoepidermal junction (Figures 2, 3). On the basis of the clinical presentation, histopathological findings, and linear IgG deposition along the dermoepidermal junction on direct immunofluorescence, a diagnosis of BP was established, while the alternative diagnoses were excluded. The patient's Bullous Pemphigoid Disease Area Index (BPDAI) activity score at presentation was 1, consistent with localized and mild disease [9]. Treatment with topical corticosteroids resulted in complete regression of the lesion within two weeks. At the one-year follow-up, no recurrence or new lesion development was observed.

## Discussion

Table 1 summarizes previously published cases of localized BP arising on surgical or procedure-related scars, including cases following incisional hernia repair, long-bone fracture fixation, limb amputation, shoulder and knee arthroplasty, laparoscopic cholecystectomy, and pacemaker placement [4-6,8,10-12], alongside the present case. Across these reports, the interval between the inciting procedure and the onset of localized BP varied widely, ranging from a few days to several decades, and the clinical course ranged from disease that remained strictly confined to the scar to cases that subsequently generalized to distant skin.

**Table 1:** Published cases of localized bullous pemphigoid developing on surgical scars or procedure sites compared with the present case. ACTH: adrenocorticotropic hormone; AZA: azathioprine; CS: corticosteroids; DIF: direct immunofluorescence; IIF: indirect immunofluorescence.

Author	Age/sex	Procedure	Latency	Clinical course	Site	Histopathology	DIF	Treatment	Outcome
MacFarlane 1989 (C1)	78/M	Incisional hernia	Decades	Localized	Abdominal scar	Subepidermal blister, eosinophils	IIF+	Prednisolone±AZA	Remission
MacFarlane 1989 (C2)	71/M	Thigh amputation	Shortly after surgery	Predominantly localized	Amputation stump	Subepidermal blister	IgG	Prednisolone	Controlled
MacFarlane 1989 (C3)	91/F	Humeral fracture scar	>20 Years	Localized	Upper arm	Subepidermal blister	IgG/C3	ACTH	Healing
Anderson 2001	79/F	Shoulder hemiarthroplasty	1 Year	Localized	Shoulder scar	Subepidermal blister	IgG/C3	Topical CS	Remission
Sen 2013	39/F	Trauma surgery	15 Years	Localized	Leg scars	Subepidermal blister	C3	Topical clobetasol	No recurrence
Lo Schiavo 2014	65/F	Hip arthroplasty	4 Months	Localized generalized	Hip wound	Subepidermal blister	IgG/C3	AZA+betamethasone	Improved
Garrido Colmenero 2015 (C1)	73/F	Laparoscopic cholecystectomy	7 Days	Localized	Periumbilical scar	Compatible	IgG/C3	Topical clobetasol	Remission
Garrido Colmenero 2015 (C2)	78/F	Pacemaker	5 Days	Localized Generalized	Pacemaker scar	Compatible	IgG/C3	Topical clobetasol	Remission
Wenzel 2021	57/F	Total knee replacement	3 Months	Localized	Knee scar	Subepidermal blister	IgG/C3	Topical betamethasone	Cleared

The majority of previously reported cases developed within days to months of the surgical procedure, and only two prior reports--an upper-arm scar following humeral fracture fixation, with a latency exceeding 20 years, and scars on the legs following trauma surgery, with a 15-year latency--approach the interval observed in our patient [5,10]. In the latter case, the authors similarly invoked Ruocco's immunocompromised district [7] to explain why koebnerization, which typically requires a much shorter interval between trauma and lesion onset, could not account for such a prolonged delay, a position echoed in a subsequent commentary on that report [13]. Our patient adds a novel dimension to this small group of exceptionally delayed cases: to our knowledge, this is the first report in which the precipitating procedure was an umbilical hernia repair, and the disease remained strictly localized to the scar with complete resolution on topical therapy alone, in contrast to several other surgically triggered cases that progressed to generalized BP [11,12].

Notably, the interval between the surgical procedure and the appearance of BP in our patient was exceptionally prolonged. This observation is consistent with Ruocco's concept of the immunocompromised district, which has been proposed as a framework that describes how a localized perturbation of the neuro-immuno-cutaneous axis may create a tissue microenvironment that is selectively susceptible to secondary immunologically mediated conditions [7]. Within such districts, an inciting disease or trauma may establish site-specific immunological alterations that render the affected area vulnerable to subsequent, unrelated disorders. Importantly, the secondary disease remains confined to the same anatomical territory and may not manifest until after a highly variable latency period [7].

Evidence from the literature suggests that a variety of external triggers may precipitate BP onset. These precipitating factors encompass physical injury, operative trauma, thermal injury from burns or radiation, and pre-existing inflammatory skin diseases such as cellulitis, lichen planus, and insect bite reactions [14-17].

The differential diagnosis of an isolated bulla on a surgical scar also includes epidermolysis bullosa acquisita, bullous allergic contact dermatitis, and bullous impetigo, each of which can present in this distribution and mimic BP on initial inspection. Epidermolysis bullosa acquisita can closely resemble BP both clinically and on direct immunofluorescence. Bullous allergic contact dermatitis typically presents with a more eczematous, intensely pruritic morphology in the setting of a recent identifiable exposure, and resolves promptly once the offending agent is withdrawn, rather than arising spontaneously years after exposure has ceased. Bullous impetigo is characterized by fragile, flaccid bullae with honey-colored crusting, usually following a preceding break in skin integrity, and responds to antimicrobial rather than immunosuppressive therapy. In our patient, the subepidermal blister on histopathology and the linear IgG pattern along the dermoepidermal junction on direct immunofluorescence, together with the absence of crusting, exposure history, or an infectious source, favored a diagnosis of BP over these alternatives [1]. A limitation of the present case is the absence of serum anti-BP180 and anti-BP230 antibody testing, as these assays were not available at our institution. Nevertheless, the diagnosis was established based on the characteristic clinical presentation, histopathological findings, and confirmatory direct immunofluorescence. In our patient, BP emerged on the scar tissue 15 years after umbilical hernia surgery, a latency period that underscores how durable these site-specific immune alterations can be.

## Conclusions

Localized BP developing over surgical scars constitutes a rare yet well-characterized entity, most parsimoniously explained through the framework of Ruocco's immunocompromised cutaneous district. The current case is distinguished by the occurrence of BP on an umbilical hernia repair scar following an exceptionally prolonged latency interval of 15 years, underscoring that site-specific immunological alterations may remain operative indefinitely.

A brief literature search was performed using the PubMed database with the keywords "bullous pemphigoid," "umbilical hernia repair," "surgical scar," and "immunocompromised cutaneous district". To the best of our knowledge, no previous reports describing delayed-onset localized bullous pemphigoid arising on an umbilical hernia repair scar were identified. Clinicians should incorporate this entity into their differential diagnosis when evaluating isolated blistering lesions overlying surgical scars, irrespective of the length of time elapsed since the procedure.
